# Evaluating Demographic Data to Improve Confidence in Equity Analytics in a Children’s Hospital

**DOI:** 10.1097/pq9.0000000000000642

**Published:** 2023-04-10

**Authors:** Anna M. Straus, Alissa Hayes, Jodi Simon, Andrea Sims, Karen Skerlong, Michele Wilmoth, Michael T. Bigham

**Affiliations:** From the * Enterprise Data and Analytics Department, Akron Children’s Hospital, Akron, Ohio; † Patient Experience Department, Akron Children’s Hospital, Akron, Ohio; ‡ Quality Services Department, Akron Children’s Hospital, Akron, Ohio; § Department of Pediatrics, Akron Children’s Hospital, Akron, Ohio; ∥ School Health Services Department, Akron Children’s Hospital, Akron, Ohio; ¶ Department of Pediatrics, Northeast Ohio Medical University, Rootstown, Ohio.

## Abstract

Healthcare institutions are placing greater emphasis on equitable care. To accurately track and validate equity metrics, Akron Children’s Hospital evaluated how key fields are collected, analyzed, and visualized throughout the organization. Standardized recommendations in this area vary, and this investigation provided specific ways to advance analytics in this field. In addition, the technical infrastructure needed a comprehensive evaluation to increase confidence in using demographic data.

**Methods:**

First, we reviewed how staff are trained to collect data at registration. Next, the electronic health record team standardized race and ethnicity fields with federal definitions. We found that fields were not consistently accessible across reporting tools. However, when present, all fields are sourced from the same electronic health record field. Finally, 6 months of encounters were analyzed and validated, with limitations to a seldom-populated Race 2 field.

**Results:**

We compared data, including and excluding null values, to provide concise recommendations for standard visualizations. We uncovered many consistencies and a few inconsistencies that informed the next steps.

**Conclusions:**

The results informed 7 recommendations to align Akron Children’s Hospital’s advancement in analytics for health equity data: standardize race and ethnicity fields across all reporting tools, add Child Opportunity Index 2.0 to the enterprise data warehouse, utilize data at the time of the patient’s encounter, include null fields (patient refused, unknown, and not specified) in analysis, increase reporting capabilities for social determinants of health (SDOH), standardize multiracial data visualizations, and optimize reliable upstream data collection to increase reliability for all health equity measures.

## INTRODUCTION

Health equity is a core tenant of healthcare quality. In 2001, the Institute of Medicine described “Six Aims for Improvement” in its influential report, *Crossing the Quality Chasm: A New Health System for the 21*^*st*^ *Century*.^1^ Healthcare institutions are emphasizing the need for equitable care to improve patient outcomes and the overall health of our communities. Professor Margaret Whitehead, head of the World Health Organization Collaborating Centre for Policy Research of the SDOH, argues that there is a moral and a business case for health equity. Health disparities lead to poorer health outcomes for disadvantaged populations. These poor outcomes increase disease incidence and costs for health systems, insurers, employers, patients, and families. Improving the overall health of these populations would not only benefit patients and families but also reduce healthcare costs.^2^

At Akron Children’s Hospital (ACH), we understand the need to improve health equity. Health equity means everyone has a fair and just opportunity to be healthier. “We were founded on the principle of serving the needs of our community. Within our doors, this means treating all children as if they were our own, treating others as they would want to be treated, and turning no child or family away.”^3^ This foundation inherently relates to equitable healthcare by ensuring “all our patients and families receive high quality, evidence-based, equitable care […]. This requires removing obstacles to health such as poverty, discrimination, and their consequences, including powerlessness and lack of access to good jobs with fair pay, quality education and housing, safe environments, and healthcare,” as noted in ACH’s Health Equity Steering Committee (HESC) Charter and resources from the Robert Woods Johnson Foundation.^4^ Forming multidisciplinary teams and improvement strategies are critical first steps. However, accessing reliable equity data for enterprise-wide use to ensure consistency in reporting and analytics is an important next step.

Collecting data necessary to track health equity measures, such as race, ethnicity, and language, is not a unique challenge to ACH.^5^ Cowden et al^6^ surveyed the practices of pediatric hospitals to assess these data. The study showed enormous variability across 93 pediatric hospitals. These hospitals captured patient demographics at a much higher rate (95%) than the parent or guardian demographics (31%), which can paint an incomplete picture of how to best serve the patient’s needs. Also, with varying data definitions of race and ethnicity across the industry and only a small percentage of hospitals (13%) offering multiracial or multiethnic registration options, healthcare institutions are further limiting the valuable information needed to inform equitable care. Furthermore, few hospitals (20%) stratify quality and safety measures by race and ethnicity or language preference when considering how to analyze and report health equity data.

We sought to evaluate and standardize key demographic fields critical to accurately reporting and analyzing if quality improvement projects positively impacted equitable care. This focus was foundational to ensure we governed demographic data from the source and reported consistently throughout ACH. In addition, centralizing our approach with all teams who built, extracted, analyzed, or reported the data aligned everyone to the same goal: providing accurate data, informing health equity decisions, and improving patient outcomes.

## METHODS

### Setting

ACH is a large freestanding children’s hospital system serving northeast Ohio with a network of 2 hospitals, 4 urgent cares, and 50 primary and specialty locations across 27 counties with over one million visits per year.

### Data Collection at Registration

Before we assessed any analyses or revision of analyst procedures, it was necessary to understand how we collect the data in Epic (Epic Systems, Verona, WI), ACH’s electronic health record (EHR). Registration staff enroll in training to collect demographic information from patients. This training to gather REaL (Race, Ethnicity, and Language) data aims to improve the quality and consistency of data. If the patient has an active Epic MyChart patient portal account, the patient or guardian may update demographic data through that application anytime. ACH also uses welcome kiosks to register patients for appointments; however, these kiosks are not available at all locations.

Training staff on demographic data collection is particularly important because they collect sensitive information. Standardizing how staff ask these questions ensures all patients experience consistent communication during registration. For example, there is a Joint Commission-recommended scripting language for staff to ask preferred language: “In what language do you prefer to discuss your healthcare” or “In what language do you prefer to discuss your child’s healthcare?” This training includes similar recommendations for race and ethnicity. To focus the scope of these efforts, our primary concern was the patient data, with the intent to follow a similar procedure for parent/guardian data.

### EHR Standardization

Akron Children’s has standardized definitions of race and ethnicity. Beginning April 1, 2020, an EHR update standardized ethnicity to match U.S. Office of Management and Budget definitions.^7^ Before this update, registration options did not align with this definition. EHR staff transferred Hispanic and Non-Hispanic patient data in the post-update environment but nullified all other ethnicity options. Thus, the patient must repopulate this information to align with the new definitions using better-governed data.

Then, on June 3, 2021, we changed the Race category to remove Hispanic, Middle Eastern Indian, and Other from the list of options a patient has at registration. The updated Race category list includes African America/Black, American Indian and Alaska Native, Asian, Native Hawaiian, and Other Pacific Islander, Patient Refused, Unknown, and White or Caucasian. We asked patients to self-identify their race according to the race options provided post-update. Before updating this field, the race definition was unclear and included some ethnicity options in this category.

### EHR Evaluation

The HESC identified 7 key fields of health equity data that are of initial focus to ACH: race, sex, ethnicity, language, zip code, payer, and patient age. These fields were selected due to their increased need for health equity reporting, so we cross-referenced them in 9 reporting tools. Table 1 illustrates the distribution of data availability in each of these reporting tools. For example, the Clinical Universe (the data mart software used internally for data reporting and analytics; SAP America, Newtown Square, PA) displays one single race category, so the data will only show the first populated option when a patient registers for a visit. However, the Patient Experience Universe (SAP America, Newtown Square, PA) displays 5 race category possibilities, so an analyst would see up to 5 races as populated at registration.

**Table 1.** Equity Measures Visible in Reporting Tools

	Equity Measures									
Reporting Tool	Race 1	Race 2*	Sex	Ethnicity	Language	Zip Code (At Encounter)†	Zip Code (Current)‡	Payer	Age (At Encounter)	Age (Current)
Clarity universes										
Appointments			✓		✓	✓	✓	✓	✓	✓
Clinical	✓		✓	✓	✓		✓	✓	✓	✓
EDW Universes§										
Medication (x2)	✓	✓	✓	✓	✓	✓	✓	✓	✓	✓
Patient experience	✓	✓	✓	✓	✓	✓	✓	✓	✓	✓
Referrals	✓	✓	✓	✓	✓	✓	✓	✓	✓	✓
Surgery (x3)	✓	✓	✓	✓	✓	✓	✓	✓	✓	✓
External universe										
Ohio Hospital Association	✓		✓	✓	✓	✓		✓	✓	
Strata										
Decision support system	✓		✓¶	✓	✓	✓		✓	✓	
QlikView data										
Patients	✓		✓	✓	✓			✓		

•The 3-letter abbreviations in the footnotes reference the Epic table where this data lives. Also referred to as the INI.

•The numbers following reference the specific field of that table. Also referred to as the item number.

• There are 3 surgery universes and 2 medication (or pharmacy) universes, as indicated in parentheses.

*If race 2 is populated (or missing), then races 3–5 match.

†Zip code at the encounter level is populated from HAR 810 (The Epic Hospital Account table and field number).

‡Zip code at the patient level is populated from EPT 80 (The Epic Generic Patient Database table and field number).

¶Strata refers to sex data from EPT 130 as “gender.” This issue is documented and will be resolved accordingly.

§This is not an exhaustive list of EDW universes. Any EDW universe not on this list will match what is demonstrated in the chart.

**Table 2.** Equity Data Sources

	Fields				
Reporting tool	Race	Sex	Ethnicity	Language	Payer
Clarity Universes					
Appointments	x	EPT (130)	x	EPT (155)	CVG (100) > EPM (.2)
Clinical	EPT (145)	EPT (130)	EPT (135)	EPT (155)	CVG (100) > EPM (.2)
EDW Universes					
Surgery (x3)	EPT (145)	EPT (130)	EPT (135)	EPT (155)	CVG (100) > EPM (.2)
Medication (x2)	EPT (145)	EPT (130)	EPT (135)	EPT (155)	CVG (100) > EPM (.2)
Referrals	EPT (145)	EPT (130)	EPT (135)	EPT (155)	CVG (100) > EPM (.2)
External Universes					
Ohio Hospital Association*	Yes	Yes	Yes	Yes	Yes
Strata					
Decision Support System	EPT (145)	EPT (130)	EPT (135)	EPT (155)	HAR (300) > CVG (110)
QlikView Data					
Patients	EPT (145)	EPT (130)	EPT (135)	EPT (155)	HAR (300) > CVG (110)

•The 3-letter abbreviations reference the Epic table where these data live. Also referred to as the INI.

•The numbers following reference the specific field of that table. Also referred to as the item number.

•There are 3 surgery universes and 2 Medication (or Pharmacy) universes as indicated in parentheses.

*The OHA universe logic is specific to the Ohio Hospital Association’s external database. These fields are all present, but cannot be traced back to Akron Children’s specific EHR fields.

After evaluating the accessibility of demographic data, the next step was determining whether each field came from the same place across the tools. Table 2 demonstrates that we source data from the same EHR field across tools except for a minor payer data discrepancy. Despite logic differences in Strata (Strata Decision Technologies, Chicago) and QlikView (Qliktech International AB, King of Prussia, PA) compared to the other tools, <2% of patient encounters resulted in different payers, and therefore, we deemed the discrepancy insignificant.

Once we confirmed the consistency of data source lineage, we pulled encounters between January 1, 2021, and June 30, 2021, to establish what percent of data was missing in Table 3. The HESC confirmed the data as most valuable when the encounter occurred to align with the patient’s circumstances when the provider delivered healthcare services. In addition, we included race 2 to reflect the usability of multiple race categories. Additional findings include a high percentage of missing payer information in the Appointment Universe. We expect this issue as we typically do not collect payer data until registration. So, the payer field will be blank if the patient does not attend the appointment, cancels the appointment, left without being seen, or has a scheduled appointment that has not yet occurred.

**Table 3.** % Data Missing from Last 6 Months of All Encounters

	Fields							
Reporting Tool	Race 1	Race 2	Gender	Ethnicity	Language	Zip Code	Payer	Age
Clarity universes								
Appointments	x	x	0	x	0	0	28	0
Clinical	6	x	0	5	0	0	6	0
EDW universes								
Medication (x2)	6	95	0	5	0	0	0	0
Surgery (x3)	6	95	0	4	0	0	0	0
Patient Experience	7	95	0	5	0	0	0	0
External universes								
Ohio Hospital Association	4	x	0	4	12	0	2	0
Strata								
Decision Support System	6	x	0	5	0	0	5	0
QlikView Data								
Patients	7	98	0	40	41	x	x	x

Fields based on the factors identified by the HESC.

% blank: blank includes: no value, unspecified, unknown, patient refused, and other.

• There are 3 surgery universes and 2 medication (or pharmacy) universes, as indicated in parentheses.

## RESULTS

**Fig. 1.**
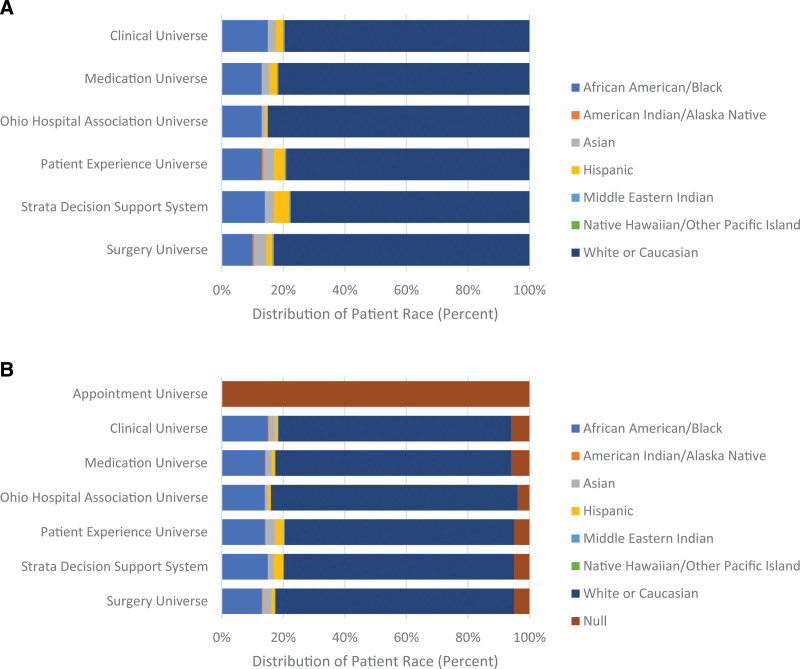
Race Distribution. A, Distribution of race across reporting tools. B, Distribution of race across reporting tools including null values. A, Six months of all encounters at Akron Children’s Hospital were included for analysis between January 1, 2021, and June 30, 2021. Race responses excluded from graph: patient refused, unknown, not specified, and blank. B, Six months of all encounters at Akron Children’s Hospital were included for analysis between January 1, 2021, and June 30, 2021. Null responses include patient refused, unknown, not specified, and blank.

Figure 1 shows the distribution of race information across reporting tools to demonstrate the impact of including null responses in a health equity query. Figure 1A shows racial distributions excluding null values, and Figure 1B shows racial distributions including null values within 6 months. This comparison is useful for evaluating which visualization the HESC recommends for use across the organization.

Standardized data collection methods, standardized definitions, consistent data sources, and inconsistent data access are the key findings that guided what next steps would be required.

## DISCUSSION

The Institute for Healthcare Improvement outlines 5 key components for healthcare organizations to improve health equity.^2^ ACH has addressed the first component to make health equity a strategic priority. The methods outlined in this article inform the second component: to develop structure and processes to support health equity work. There remain several opportunities to improve demographic data and health equity analytics. Continued data governance efforts must focus on standardizing the data available in the reporting tools, standardizing the analytics approach and visualization to correctly communicate the data, and enhancing the organizational understanding of ACH’s opportunities and shortcomings.

Before this investigation, it was unknown if users had consistent access to demographic fields across reporting tools. Standardizing the data available is a foundational step to advancing Akron Children’s capabilities in providing equitable care. Currently, there is a lack of confidence that some fields, such as race and multiracial patient demographics, are complete and accurate. Ninety-three pediatric hospitals in the United States and Canada reported similar challenges.^6^ This study reported only 13% of hospitals offered multiracial options at registration. Akron Children’s does offer patients the option to submit multiple races; however, the ability to report on that field is limited. Ensuring all reporting tools are standardized enables all stakeholders to retrieve the same data.

**Fig. 2.**
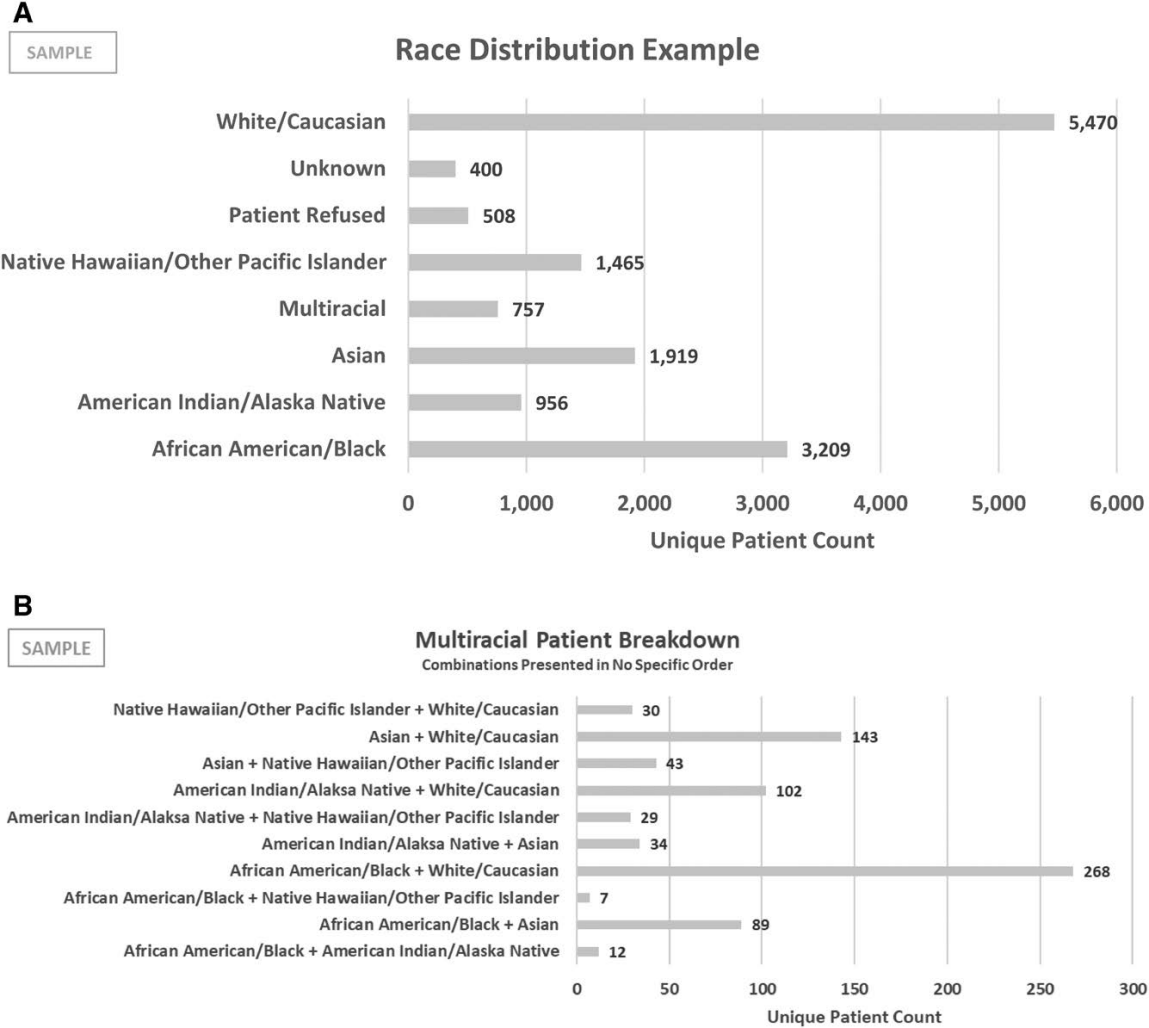
Race and Multirace Categories. A, Recommended race distribution visualization. B, Recommended multiracial patient breakdown visualization. A, This chart demonstrates the first step in visualizing multiracial patient data. First, report singular race attribution with a multiracial aggregate. B, This chart demonstrates the second step in visualizing multiracial patient data. Second, report a detailed multiracial patient breakdown with all race combinations.

After establishing this data foundation, the next step is to provide standard processes for reporting and analytics.^8^ Impactful data storytelling is only possible if we translate demographics required for measuring equity progress into usable, visible information. The inclusion criteria and combinations for reporting multiracial patients vary at present. Akron Children’s HESC provided guidance on responses in all data visualizations, including Unknown or Patient Refused. The HESC designed a standard for grouping and visualizing patients by race, with guidelines on multiracial patients (Figure 2). Cowden’s study found that only 20% of hospitals stratify quality and safety measures by race and ethnicity. Although ACH does stratify some quality and safety measures by patient race and ethnicity, reporting and analytics standards will transition our organization from unique and individual data requests, including these fields, to normalizing equity analytics. Governing how best to influence reporting standards and visualizations will help ACH achieve IHI’s second component to improve health equity by improving trust and confidence data used to make impactful decisions.

Finally, once we shape the data and analytics framework, this data must be actionable. Increased organizational understanding of health equity definitions and demographics has furthered ACH’s interest in validated best practices like SDOH and child opportunity index (COI)^9^ to improve the disproportionate morbidity felt within neighborhoods characterized by lower educational, environmental, and socioeconomic opportunity.^10^ Unweaving the complexity of these demographic fields brought to attention the lack of valuable tools like the COI and the underutilization of others, like SDOH surveys. Although SDOH surveys are currently used in a few specialties, expanding on this and building COI into regular workflows can more broadly reflect a patient’s obstacles in health and give leaders and providers a clearer picture of improvement opportunities. Using these demographic fields to stratify organizational metrics, especially those of strategic importance, is critical in pursuing health equity.

Our journey revealed the following 7 recommendations:

1. Tools
 a. Standardize race and ethnicity across all reporting tools to ensure consistent and trustworthy health equity analytics.
 b. Add COI 2.0 to the enterprise data warehouse to gather a more comprehensive view of the patient’s equity challenges.
2. Reporting and Visualization
  a. Utilize data at the patient’s encounter to standardize reporting the patient’s circumstances when the provider delivered healthcare services.
  b. Include null fields (Patient Refused, Unknown, Not Specified) in reporting, analytics, and data visualization.
  c. Increase reporting capabilities for SDOH in all specialties.
  d. Standardize multiracial data visualizations in 2 ways:
  i. 1st: singular race attribution with multiracial aggregate (Fig. 2A)
  ii 2nd: detailed multiracial patient breakdown with all race combinations (Fig. 2B)
3. Operations
  a. Optimize reliable upstream data collection to further trust and reliability in data.

To measure the success of this analytics investment, Akron Children’s will target all data requests that come in through our service request process, and 90% of completed requests will have health equity data formatted according to these recommendations. Additionally, aiming for 75% of ACH-led publications and abstracts to report equity domains will emphasize the integration of equitable care into the foundation of how ACH provides care. To successfully improve upon these, baseline data will be tracked and reported. ACH is applying equity analytics to several clinical outcomes, including completed well-check visits, reducing nil per os (nothing per mouth) violations before surgery, focused care for suicide risk patients, vaccination compliance, and improving asthma and diabetes outcomes.

Healthcare technology, infrastructure, and healthcare services can move rapidly. Therefore, ensuring this evaluation is complete is pivotal if future quality improvement projects want to use reliable data to support health equity measures. Akron Children’s staff have already completed extensive work but in silos. Uncovering and sharing this groundwork can accelerate the development of metrics and reports for health equity projects.

There are some limitations of this investigation. First, the tools and technical infrastructure in place may be unique to ACH. Other organizations may use different tools but can apply similar methodology in other EHR systems and related analytics tools. Second, this investigation was limited to 7 prioritized fields, though additional fields could be evaluated using a similar process in the future. Third, beyond our investigation’s scope, upstream data collection accuracy is necessary for all the subsequent data and analytics described in this study.

W. Edwards Deming once said, “You can’t manage what you don’t measure.” Health equity is a core tenant of healthcare quality, and understanding the measurement of equity is critical to managing and improving the health outcomes of our community. We have demonstrated a process for evaluating data fields and core resources and recommended standard reporting conventions using these key health equity fields. We recommend organizations committed to achieving health equity undertake this same disciplined evaluation at their organization.

## ACKNOWLEDGMENTS

I want to acknowledge the contributions of Gary Cao for his leadership on the content needed to evaluate our data and analytics capabilities and future direction. Thanks to Carole Becerra and Heidi Perris for guidance on current registration practices and training as well as diversity, equity, and inclusion direction, from Samantha Formica, Terri Lowe-Donovan, and Ashley Morris on current population health and managed care needs, from Janice Juszczec and Sandy Brotje for technical background and education on the flow of data through systems, from Adam Zimmerman on the Epic background for how we capture these measures, and from Dr. Joel Davidson and Amanda Patterson on current efforts to capture and analyze social determinants of health. These contributions provided a significant background for this investigation and helped frame the recommendations for Akron Children’s Hospital’s journey for advancement in health equity.

## DISCLOSURE

The authors have no financial interest to declare in relation to the content of this article.

## References

[1] Institute of Medicine (US) Committee on Quality of Health Care in America. Crossing the Quality Chasm: A New Health System for the 21st Century. National Academies Press. Available at http://www.ncbi.nlm.nih.gov/books/NBK222274/. 2001. Accessed April 6, 2022. 25057539

[2] Wyatt R Laderman M Botwinick L. Achieving Health Equity: A Guide for Health Care Organizations. IHI White Pap. Available at http://www.ihi.org/resources/Pages/IHIWhitePapers/Achieving-Health-Equity.aspx. 2016. Accessed April 7, 2022.

[3] Akron Children’s Hospital. About Akron Children’s Hospital. Available at https://www.akronchildrens.org/pages/About-Us.html. 2022. Accessed April 7, 2022.

[4] Braveman P Arkin E Orleans T. What Is Health Equity? Robert Woods Johnson Foundation. Available at https://www.rwjf.org/en/library/research/2017/05/what-is-health-equity-.html. 2017.

[5] Williams JS Walker RJ Egede LE. Achieving equity in an evolving healthcare system: opportunities and challenges. Am J Med Sci. 2016;351:33–43. 26802756 10.1016/j.amjms.2015.10.012 PMC4724388

[6] Cowden JD Flores G Chow T. Variability in collection and use of race/ethnicity and language data in 93 pediatric hospitals. J Racial Ethn Health Disparities. 2020;7:928–936. 32056162 10.1007/s40615-020-00716-8

[7] Office of Management and Budget. Revisions to the standards for the classification of federal data on race and ethnicity. Fed Regist. Available at https://www.govinfo.gov/content/pkg/FR-1997-10-30/pdf/97-28653.pdf. 1997;62 (No. 210). Accessed April 7, 2022.

[8] Kilbourne AM Switzer G Hyman K. Advancing health disparities research within the health care system: a conceptual framework. Am J Public Health. 2006;96:2113–2121. 17077411 10.2105/AJPH.2005.077628 PMC1698151

[9] diversitydatakids.org. Child Opportunity Index (COI). Diversitydatakids.org. Available at https://www.diversitydatakids.org/child-opportunity-index. 2022. Accessed April 7, 2022.

[10] Krager MK Puls HT Bettenhausen JL. The child opportunity index 2.0 and hospitalizations for ambulatory care sensitive conditions. Pediatrics. 2021;148:e2020032755. 34215676 10.1542/peds.2020-032755

